# Metabolic signature of short‐term low energy availability

**DOI:** 10.14814/phy2.70582

**Published:** 2025-09-29

**Authors:** Valentin Nusser, Chaise Murphy, Sieglinde Hechenbichler Figueroa, Alexander Braunsperger, Johanna K. Ihalainen, Juha J. Hulmi, Paulina Wasserfurth, Karsten Koehler

**Affiliations:** ^1^ Professorship of Exercise, Nutrition and Health, TUM School of Medicine and Health Technical University of Munich Munich Germany; ^2^ School of Health and Human Sciences South Dakota State University Brookings South Dakota USA; ^3^ Professorship of Exercise Biology, TUM School of Medicine and Health Technical University of Munich Munich Germany; ^4^ Faculty of Sport and Health Sciences University of Jyväskylä Jyväskylä Finland; ^5^ Finnish Institute of High Performance Sport KIHU Jyväskylä Finland; ^6^ Deparment of Nutrition and Health Sciences University of Nebraska‐Lincoln Lincoln USA

**Keywords:** energy metabolism, low energy availability, metabolomics

## Abstract

Exposure to low energy availability (LEA) can potentially detrimentally affect athletes' health and performance. Timely identification is crucial, yet its detection is often delayed until severe symptoms emerge. Our objective was to identify characteristic differences in the serum metabolome as potential early LEA biomarkers. We performed large‐scale metabolomics analyses of data from two highly controlled, randomized controlled trials, exposing trained adults to short‐term (3–5 days) low or high energy availability (15 (LEA) versus 40 (HEA) kcal·kg FFM^−1^·day^−1^), which were achieved once with and once without daily aerobic exercise. Differences between LEA and HEA were prominent in triglycerides (0.66 ± 0.22 vs. 1.07 ± 0.47 mmol·L^−1^), total fatty acids (9.46 ± 1.50 vs. 11.22 ± 2.59 mmol·L^−1^), amino acids (e.g., alanine: 0.46 ± 0.10 vs. 0.58 ± 0.15 mmol·L^−1^), very‐low‐density lipoproteins (0.57 ± 0.23 vs. 0.67 ± 0.25 mmol·L^−1^), and ketone bodies (e.g., β‐hydroxybutyrate: 364 ± 241 vs. 30 ± 17 μmol·L^−1^; all FDR < 0.05). These patterns reveal a marked shift towards increased fat utilization, altered lipoprotein profiles, and enhanced ketogenesis in response to short‐term LEA. Post‐intervention β‐hydroxybutyrate (>0.09 mmol·L^−1^) best predicted LEA, regardless of whether LEA was achieved with or without exercise, supporting its candidacy for LEA screening. Overall, our findings provide new insight into the metabolomic signature of LEA and support metabolomics as a tool for early detection of LEA.

## 1 INTRODUCTION

Maintaining adequate energy availability (EA) to ensure optimal health and performance is a key consideration for athletes (Wasserfurth et al., 2020). EA is defined as the dietary energy that remains for all physiological functions after subtracting exercise energy expenditure (EEE) from energy intake (EI) (Areta et al., 2021). Low EA (LEA), the underlying cause of the Female Athlete Triad (Nattiv et al., 2007) and Relative Energy Deficiency in Sport (REDs) (Mountjoy et al., 2023), is associated with potentially detrimental effects on health (Logue et al., 2018) and performance (Melin et al., 2019, 2024). The impact of LEA on metabolic and endocrine pathways is well documented in current literature and includes key regulators of energy metabolism such as leptin and triiodothyronine (Koehler et al., 2016; Loucks & Heath, 1994) and markers of reproductive function (Loucks, 2020), bone turnover (Ihle & Loucks, 2004; Murphy et al., 2021; Papageorgiou et al., 2017, 2018), and iron metabolism (Ishibashi et al., 2020).

The screening for signs of LEA, as has traditionally relied on the assessment of clinical symptoms, such as amenorrhea (Loucks & Thuma, 2003) or bone stress injuries (Mountjoy et al., 2023; Sterringer & Larson‐Meyer, 2022). However, clinical symptoms often manifest as a long‐term consequence of LEA, delaying both its identification and management. On the other hand, questionnaires have emerged as more practical tools for the assessment of athletes at risk of LEA (Torstveit et al., 2023). Yet, these instruments typically focus on identifying the presence of aforementioned clinical symptoms or problematic behaviors that may increase the risk of LEA, and their efficacy in accurately distinguishing athletes at risk of LEA is a topic of ongoing debate (Sim & Burns, 2021; Stellingwerff et al., 2023).

As a result, there is an increasing interest in the identification of early biomarkers of LEA, which could facilitate timely detection and prevention of long‐term health consequences. In this context, metabolomics, the comprehensive analysis of metabolites within a biological specimen, emerges as a novel and promising approach for discovering potential new biomarkers and understanding the complex and unique metabolic manifestations associated with LEA, that is, its metabolic signature (Monteiro et al., 2013; Roessner & Bowne, 2009; Rubio‐Aliaga et al., 2010). Metabolomics enables the detection of condition‐specific metabolic alterations, which could lead to the identification of biomarkers that can serve as an early warning system, enabling interventions before the onset of more severe and irreversible health consequences (Melin et al., 2019; Wasserfurth et al., 2020).

We performed a large‐scale analysis of blood metabolites and metabolite ratios in two cohorts of trained adults under highly controlled laboratory conditions (Murphy et al., 2021; Murphy & Koehler, 2020). We employed nuclear magnetic resonance (NMR) spectroscopy, which offers a selective yet highly reproducible and quantitatively accurate profile of the metabolome. It is particularly suited for detecting changes in lipid‐related and low‐molecular‐weight metabolites (Letertre et al., 2021), which are likely to occur in response to energetic challenges such as LEA (Jouhki et al., 2024). Furthermore, NMR's fast and robust metabolomic analysis makes it an especially suitable candidate for routine biomarker detection.

Our investigation was structured around several objectives. First, we sought to identify biomarkers that quickly respond to a short (3–5 days) and controlled induction of LEA. Based on observations in free‐living physique athletes during competition preparation, which typically involves exposure to LEA for reducing body weight and fat mass (Jouhki et al., 2024), we hypothesized that short‐term exposure to LEA would induce similar characteristic changes in the human metabolome, favoring fat utilization and ketogenesis. Next, we examined post‐intervention samples to characterize the metabolic profile relevant for practical LEA screening. We further hypothesized that LEA induces characteristic metabolic alterations, although some of these changes may be modulated by exercise. Thus, our goal was to identify metabolites that vary with differing levels of energy availability and determine which are affected by the presence or absence of exercise. Finally, we assessed the capacity of the metabolic signature to predict LEA status.

## 2 MATERIALS AND METHODS

### 2.1 Study design

**FIGURE 1:**
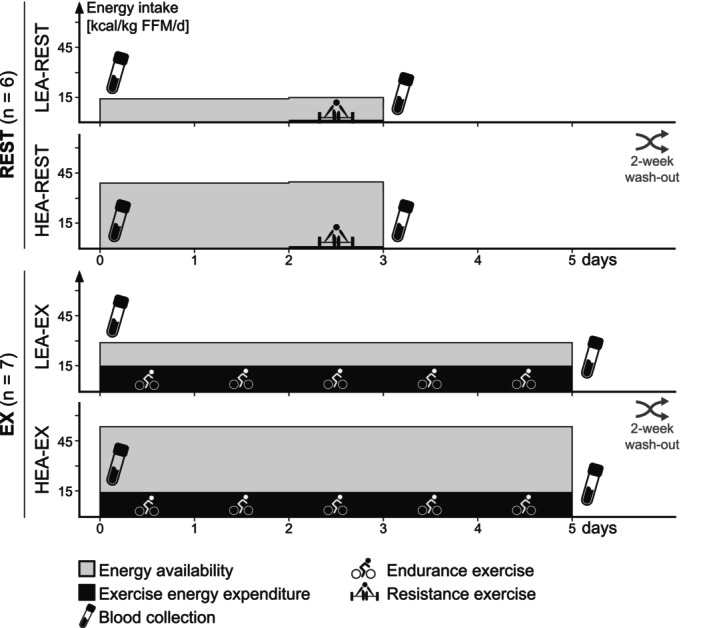
Study designs of REST and EX as well as for each high (HEA‐REST and HEA‐EX) and low energy availability (LEA‐REST and LEA‐EX) condition. Fasted serum blood samples were collected before and right after the interventions.

The present analysis combines data sourced from two published clinical trials (Murphy et al., 2021; Murphy & Koehler, 2020). Both studies were randomized, single‐blind, repeated‐measures crossover trials designed to assess associations between dietary interventions and metabolic alterations during short‐term LEA. For the present analysis, we selected data from each study for one condition in which EA was reduced to 15 kcal·kg FFM^−1^·day^−1^ (LEA), and one control condition in which EA was kept at 40 kcal·kg FFM^−1^·day^−1^ (high energy availability; HEA). A reduction of EA to 15 kcal·kg FFM^−1^·day^−1^ has previously been shown to result in significant metabolic perturbations during comparable short‐term interventions (Ihle & Loucks, 2004; Kojima et al., 2020; Loucks & Heath, 1994; Papageorgiou et al., 2017). In order to account for a potential modulating effect of exercise, we selected data from one study in which LEA was achieved by simultaneously restricting EI and increasing EEE (EX) (Murphy et al., 2021), and from another study in which LEA was achieved by restricting EI only (i.e., no meaningful exercise expenditure; REST) (Murphy & Koehler, 2020). Participants in EX engaged in daily aerobic exercise to expend 15 kcal·kg FFM^−1^·day^−1^, while participants in REST abstained from exercise with the exception of a single bout of resistance training on the last intervention day. In both studies, participants underwent conditions in a random order and completed a washout period of at least 2 weeks between conditions during which they resumed habitual exercise and dietary practices (Figure 1). The intervention duration was 5 days for EX and 3 days for REST. Both studies were approved by the University of Nebraska Institutional Review Board (IRB#: 20160315895FB, IRB#: 20180617933FB), conducted according to the Declaration of Helsinki, and all participants provided their written informed consent, including permission for the use of their samples in future exploratory analyses.

### 2.2 Participants

For both studies, we recruited participants from campus and other local recreation sites via flyers, emails to campus sports clubs, and social media posts. Full recruitment with inclusion and exclusion of participants was previously described (Murphy et al., 2021; Murphy & Koehler, 2020). To minimize training effects, both studies included participants who were familiarized with aerobic exercise (EX) or resistance exercise (REST).

### 2.3 Study procedures

In the morning of the first and after the last day of each condition (Figure 1), following an overnight fast of at least 10 h, participants reported to the laboratory for the assessment of body composition and blood sampling. Fasted blood samples were collected and stored as serum aliquots at −80°C until analysis. Participants in EX also completed a graded exercise test on a cycle ergometer (LC6, Monark HB, Vansbro, Sweden), as daily exercise intensity was prescribed relative to peak oxygen consumption (V̇O_2peak_). Final exercise bouts were conducted at least 16–22 h prior to blood collection at POST.

### 2.4 Diet preparation

Participants received all food consumed during each condition in liquid form. Diets consisted of an individually tailored combination of clinical products (Ensure Plus; 4.6 g protein·100 kcal^−1^ and Ensure High Protein; 10 g protein·100 kcal^−1^, both Abbott Nutrition, Chicago, IL, USA), maltodextrin (Tate and Lyle, London, UK), and whey protein isolate (unflavored, Isopure, IL, USA). In EX, participants consumed either 30 kcal·kg FFM^−1^·day^−1^ (LEA‐EX) or 55 kcal·kg FFM^−1^·day^−1^ (HEA‐EX), and protein intake was maintained at 1.7 g·kg BW^−1^·day^−1^ in both conditions. In REST, participants consumed either 15 kcal·kg FFM^−1^·day^−1^ (LEA‐REST) or 40 kcal·kg FFM^−1^·day^−1^ (HEA‐REST), and protein intake was maintained at 1.2 g·kg BW^−1^·day^−1^ in both conditions. Throughout all conditions, participants were permitted water consumption ad libitum, but other beverages were prohibited. During EX, participants received adequate fluid (800 mL water·h^−1^ exercise with 1.2 g sodium chloride·L^−1^) to prevent dehydration (Sawka et al., 2007).

### 2.5 Exercise prescription

In EX, participants completed daily supervised sessions of aerobic exercise on a cycle ergometer at the power output corresponding to 60% of their V̇O_2peak_. The duration of the daily exercise sessions was calculated by dividing the target energy expenditure of the exercise session (15 kcal·kg FFM^−1^·day^−1^) by the rate of energy expenditure at 60% V̇O_2peak_. Additional exercise and intense physical activity were prohibited. In REST, participants did not perform any exercise with the exception of a single resistance exercise session consisting of five sets of five repetitions of barbell back squats with at least one repetition in reserve, which participants conducted on the final day (Murphy & Koehler, 2020). Compliance was measured via a waist‐worn accelerometer (ActiLife G3TX+, ActiGraph, Pensacola, FL, USA).

### 2.6 Metabolomics

Serum samples were analyzed for 250 outcome variables (165 metabolites, 82 metabolite ratios, and three lipoprotein particle sizes) using high‐throughput quantitative NMR spectroscopy (Soininen et al., 2015; Würtz et al., 2017) at a commercial laboratory (Nightingale Health Plc, Helsinki, Finland). Metabolites included lipids, lipoprotein subclass profiling with lipid concentrations within 14 subclasses, fatty acid composition, inflammation marker glycoprotein acetyls (GlycA), and various low‐molecular metabolites including amino acids, ketone bodies, and gluconeogenesis‐related metabolites. Metabolite ratios included fatty acid ratios and relative lipoprotein lipid concentrations. Original metabolomics data were imputed (k‐nearest neighbors), log_2_‐transformed, and pareto‐scaled using a free‐to‐use online platform (VIIME) (Choudhury et al., 2020) in order to reduce skewness and the relative magnitude of large values. Prior to statistical analysis, variables that were measured below the limit of quantification for more than 10% of all observations were excluded from further analysis.

### 2.7 Statistical analyses

All statistical operations were performed in R (version 4.3.2, R Foundation for Statistical Computing, Vienna, Austria). We evaluated between‐study and within‐study differences in body composition and dietary EI using unpaired and paired Student's *t*‐tests, respectively. We then analyzed changes in metabolite concentrations from pre‐ to post‐intervention using Generalized Estimating Equations (GEE) with Gaussian family, linear link function, and independent correlation structure. In order to investigate LEA's metabolic signature in comparison to the control condition, we also employed GEE models using post‐intervention metabolite concentrations only. Changes were modeled with fixed effects for time (pre vs. post), condition (LEA vs. HEA), state (EX vs. REST), and their interactions, as applicable. Post‐intervention differences were modeled with fixed effects for condition, state, and their interaction. In all models, “condition” denotes the dietary condition (LEA vs. HEA), and “state” denotes the intervention context (EX vs. REST). We adjusted all *p* values for multiple testing using the Benjamini‐Hochberg correction (false discovery rate, FDR). Finally, imputed but not transformed metabolite concentration values were each included in a univariate logistic regression model to assess their individual capacity to predict LEA status with the potential to serve as possible biomarker(s) of LEA. In the next step, we included those variables with the highest predictive power, starting with the top 25 features ranked by univariate model performance, in a multivariable logistic regression model. In a stepwise manner, we reduced the number of features introduced into the logistic regression model until the Akaike information criterion (AIC) was at its lowest in order to prevent overfitting (Grissa et al., 2016).

## 3 RESULTS

In total, data from seven participants who completed EX (all male) and six participants who completed REST (including two females) were included in the final analysis. Participants in EX and REST were comparable in age (24 ± 4 vs. 22 ± 3 years) as well as baseline weight (85.4 ± 7.7 vs. 75.6 ± 17.4 kg), FFM (70.4 ± 7.1 vs. 62.1 ± 15.7 kg), and body fat percentage (17.6 ± 3.3 vs. 18.6 ± 7.2%). Per study design, EA differed between LEA and HEA (*p* < 0.001), but it was not different between EX and REST for LEA (14.8 ± 1.4 vs. 15.3 ± 0.3 kcal·kg FFM^−1^·day^−1^) nor HEA (40.1 ± 2.9 vs. 40.7 ± 1.3 kcal·kg FFM^−1^·day^−1^). Therefore, there was similar weight change between LEA‐EX (−0.45 ± 0.27 kg·d^−1^) and in LEA‐REST (−0.64 ± 0.12 kg·d^−1^; *p* = 0.14). On the other hand, due to the increased EEE in EX, EI as well as carbohydrate and fat intakes were higher in EX when compared to REST (*p* < 0.05; Table 1).

**TABLE 1:** Energy intake, expenditure, and availability.

	LEA		HEA		
	EX (*n* = 7)	REST (*n* = 6)	EX (*n* = 7)	REST (*n* = 6)	
EI [kcal/kg FFM/d]	31.1 ± 1.4	15.2 ± 0.6**	56.4 ± 2.5	40.7 ± 1.3**	^#^
CHO [g/kg/d]	3.3 ± 0.2	1.5 ± 0.2**	7.8 ± 0.8	4.9 ± 0.5**	^#^
Fat [g/kg/d]	0.3 ± 0.1	0.2 ± 0.0**	1.3 ± 0.1	1.0 ± 0.1**	^#^
Protein [g/kg/d]	1.6 ± 0.4	1.2 ± 0.1*	1.7 ± 0.0	1.2 ± 0.1*	
EEE [kcal/kg FFM/d]	16.4 ± 1.0	n/a	16.2 ± 2.1	n/a	
EA [kcal/kg FFM/d]	14.8 ± 1.4	15.2 ± 0.6	40.1 ± 2.9	40.7 ± 1.3	^#^
CHO oxidation during exercise [g/kg/d]	2.4 ± 0.6	n/a	2.1 ± 0.4	n/a	
CHO availability [g/kg/d]	0.9 ± 0.5	1.5 ± 0.2*	5.2 ± 1.0	4.9 ± 0.5	^#^

*Note*: Values are displayed as mean ± standard deviation. Significant difference between conditions: ^#^ *p* < 0.001; significant difference between states: **p < 0.001, *p < 0.05.

Abbreviations: CHO, carbohydrate; EA, energy availability; EEE, exercise energy expenditure; EI, energy intake; FFM, fat‐free mass.

For the metabolomics analysis, 16 out of 250 variables fell below the limit of quantification in ≥10% of observations and were subsequently excluded from further analyses. Further, due to the unavailability of pre‐intervention blood samples for a subset of our cohort, one participant from EX and two participants from REST (one female and one male) were omitted from the comparative analysis of pre‐ and post‐intervention data.

### 3.1 Changes in metabolome in response to short‐term LEA

**FIGURE 2:**
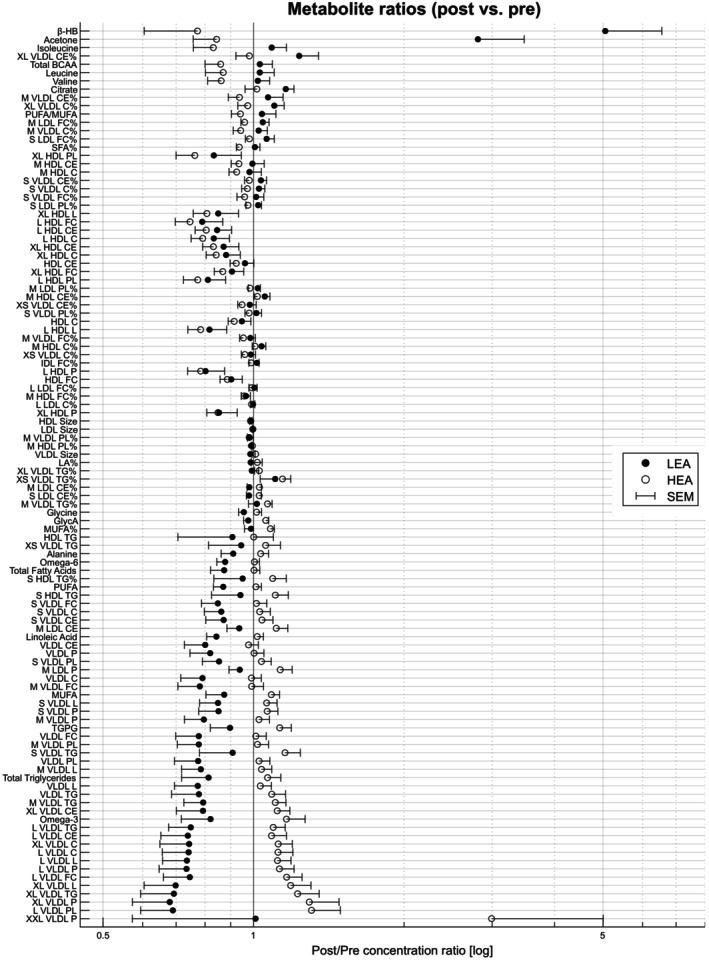
Ranked mean metabolite change‐ratios for low and high energy availability (LEA vs. HEA) for all metabolites, which showed a significant time × condition interaction effect (FDR < 0.05). Lipoprotein names include particle sizes (XXL, XL, L, M, S, and XS), lipoprotein class declarations (HDL, high‐density lipoprotein; IDL, intermediate density lipoprotein; LDL, low‐density lipoprotein; VLDL, very‐low‐density lipoprotein) and the indication of particle components (C, cholesterol; CE, cholesteryl esters; FC, free cholesterol, L, total lipids, P, concentration; PL, phospholipids; TG, triglycerides). “%” indicates a ratio of the respective component to total lipids. BCAA, branched‐chain amino acids; FA, fatty acids; LA, linoleic acids; MUFA, monounsaturated FA; PUFA, polyunsaturated FA; SEM, standard error of the mean; SFA, saturated FA; TG/PG, ratio of TG and phosphoglycerides; β‐HB, β‐hydroxybutyrate.

The refined dataset (*N* = 10) revealed a significant interaction effect between the timing of the sample collection (pre‐ vs. post‐intervention) and condition (LEA vs. HEA) for 75 out of 234 outcome variables (FDR < 0.05). Pre‐post changes pooled across EX and REST are shown in Figure 2 and stratified results for EX and REST are provided in Figures S1 and S2, respectively.

Specifically, when compared to HEA, there was a greater reduction in triglycerides (−302 ± 394 vs. 33 ± 218 μmol·L^−1^) and total fatty acids as the sum of all esterified fatty acids within serum lipoproteins (−1.29 ± 1.53 vs. 0.03 ± 1.01 mmol·L^−1^) in LEA (both FDR < 0.05; Figure 2). A decline in LEA was also observed for alanine (−56 ± 91 vs. 18 ± 74 μmol·L^−1^; FDR < 0.05) and GlycA (−44 ± 65 vs. 45 ± 32 μmol·L^−1^; FDR < 0.05), indicating a broader impact on metabolic pathways. Conversely, analysis revealed an increase in the ketone bodies β‐hydroxybutyrate (β‐HB; 309 ± 255 vs. −12 ± 19 μmol·L^−1^; FDR < 0.001) and acetone (50 ± 41 vs. −3 ± 4 μmol·L^−1^; FDR < 0.001) in LEA compared to HEA. Total branched‐chain amino acids (BCAA) decreased in HEA (−104 ± 173 μmol·L^−1^) when compared to LEA (14 ± 136 μmol·L^−1^; FDR < 0.001).

Distinct alterations in the lipoprotein profile were particularly notable in VLDL, in which particle size decreased in LEA (−0.68 ± 0.74 nm) but increased in HEA (0.37 ± 0.34 nm; FDR < 0.05). A similar observation was made for triglycerides (−248 ± 321 vs. 34 ± 156 μmol·L^−1^), free cholesterol (−59 ± 60 vs. 1 ± 38 μmol·L^−1^), phospholipids (−102 ± 108 vs. 5 ± 62 μmol·L^−1^), and total lipids (−0.48 ± 0.53 vs. 0.03 ± 0.27 mmol·L^−1^; all FDR < 0.05) in VLDL, which decreased in LEA but did not change in HEA. Serum concentrations of VLDL (−132 ± 134 vs. −10 ± 97 μmol·L^−1^) and cholesteryl esters (−73 ± 74 vs. −11 ± 59 μmol·L^−1^) also showed greater reductions in LEA when compared to HEA (both FDR < 0.05). These differential changes are present throughout all VLDL subclasses, except XS‐VLDL. Triglycerides decreased throughout all lipoprotein subclasses, although not statistically significant for some of them.

### 3.2 LEA‐induced metabolic signature is predominantly robust against the influence of exercise

Upon evaluating post‐intervention (*N* = 13) metabolite concentrations and metabolite ratios, significant main effects attributed to condition were found in 103 metabolites. Of these, the vast majority (81%) were independent of whether LEA was induced in the absence (REST) or presence (EX) of exercise, and only 20 metabolites showed a notable interaction between condition and state (FDR < 0.05).

Differences in post‐intervention serum metabolite concentrations between LEA and HEA were especially prominent in triglycerides (0.66 ± 0.22 vs. 1.07 ± 0.47 mmol·L^−1^; FDR < 0.001), total fatty acids (9.46 ± 1.50 vs. 11.22 ± 2.59 mmol·L^−1^; FDR < 0.05), the amino acids alanine (0.46 ± 0.10 vs. 0.58 ± 0.15 mmol·L^−1^; FDR < 0.001), glutamine (0.50 ± 0.09 vs. 0.49 ± 0.11 mmol·L^−1^; FDR < 0.05), and isoleucine (0.08 ± 0.02 vs. 0.07 ± 0.02 mmol·L^−1^; FDR < 0.05), pyruvate (47 ± 26 vs. 58 ± 17 μmol·L^−1^; FDR < 0.05), glycerol (182 ± 79 vs. 220 ± 152 μmol·L^−1^; FDR < 0.05), ketone bodies, and metabolites related to the VLDL lipoprotein profile.

**FIGURE 3:**
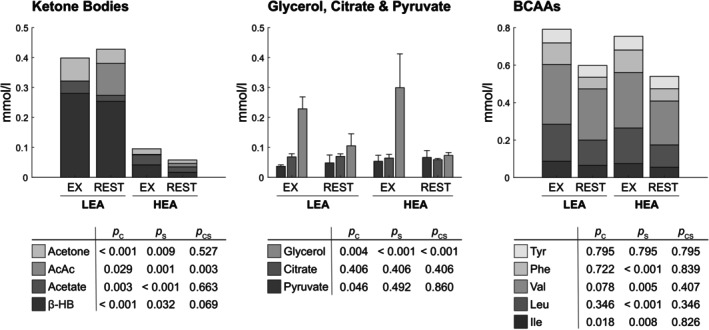
Bar plots displaying post‐intervention ketone body, glycolysis‐related metabolite, and branched‐chain amino acid concentrations for low and high energy availability (LEA vs. HEA), with and without exercise (LEA‐EX, HEA‐EX, LEA‐REST, and HEA‐REST). FDR‐corrected *p* values indicate effects of condition (*p*_C_), state (*p*_S_), and their interaction (*p*_CS_). AcAc, acetoacetate; BCAA, branched‐chain amino acid; Ile, isoleucine; Leu, leucine; Phe, phenylalanine; Tyr, tyrosine; Val, valine; β‐HB, β‐hydroxybutyrate.

Markers of ketosis, such as β‐HB (364 ± 241 vs. 30 ± 17 μmol·L^−1^; FDR < 0.001), acetate (34 ± 15 vs. 28 ± 13 μmol·L^−1^; FDR < 0.05), acetoacetate (69 ± 106 vs. 6 ± 7 μmol·L^−1^; FDR < 0.05), and acetone (74 ± 37 vs. 14 ± 2 μmol·L^−1^; FDR < 0.001) demonstrated significantly higher concentrations in LEA (Figure 3). Most of these ketone concentration differences between LEA and HEA were robust against the presence of exercise, except for acetoacetate, which was not detectable in EX. Serum pyruvate concentrations were not different between EX and REST, but glycerol concentrations were overall higher in EX when compared to REST (FDR < 0.001). More specifically, concentrations of circulating glycerol were higher in LEA‐EX compared to LEA‐REST (239 ± 33 vs. 96 ± 18 μmol·L^−1^) and in HEA‐EX compared to HEA‐REST (318 ± 111 vs. 71 ± 10 μmol·L^−1^, FDR < 0.001; Figure 3). Out of all amino acids, exercise modulated the effect of LEA only for glutamine (FDR < 0.05), which was lower in LEA‐EX when compared to LEA‐REST (0.45 ± 0.05 vs. 0.60 ± 0.02 mmol·L^−1^) and in HEA‐EX compared to HEA‐REST (0.41 ± 0.06 mmol·L^−1^ vs. 0.60 ± 0.06 mmol·L^−1^).

**FIGURE 4:**
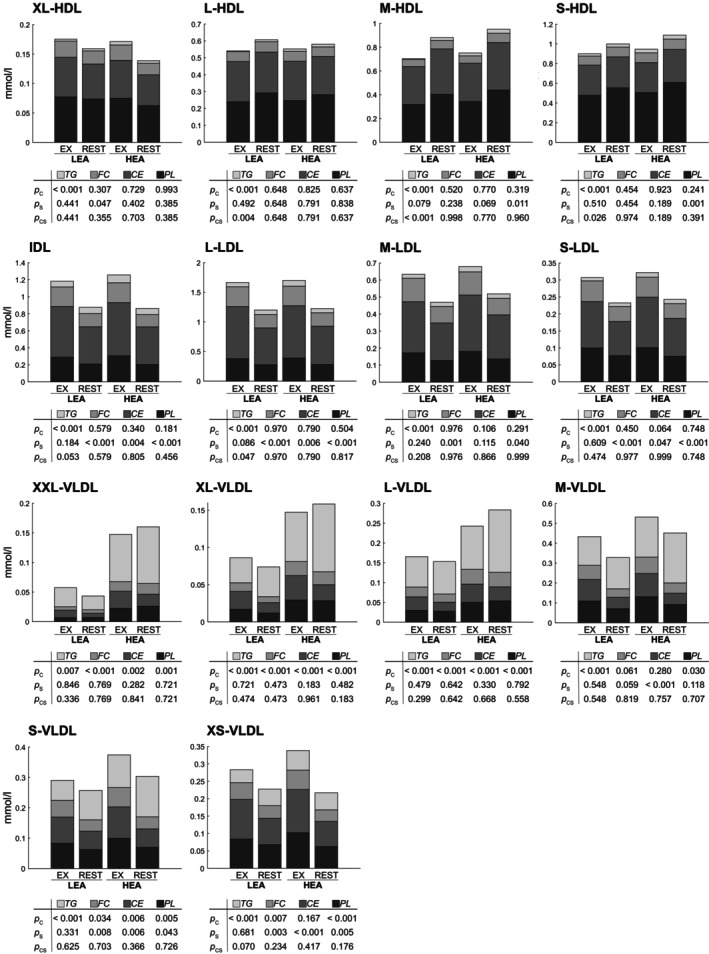
Stacked bar‐plots displaying post‐intervention serum lipoprotein concentrations of different lipoprotein subclasses for low and high energy availability (LEA vs. HEA), with and without exercise (LEA‐EX, HEA‐EX, LEA‐REST, and HEA‐REST). FDR‐corrected *p* values indicate effects of condition (*p*_C_), state (*p*_S_), and their interaction (*p*_CS_). Lipoprotein names include particle sizes (XXL, XL, L, M, S, and XS), lipoprotein class declarations (HDL, high‐density lipoprotein; IDL, intermediate density lipoprotein; LDL, low‐density lipoprotein; VLDL, very‐low‐density lipoprotein), and the indication of particle components (CE, cholesteryl esters; FC, free cholesterol; PL, phospholipids; TG, triglycerides). Numerical metabolite concentrations are provided in Table S1.

The average diameter of VLDL particles (37.6 ± 0.8 vs. 38.6 ± 1.4 nm; FDR < 0.001), VLDL serum concentration (0.57 ± 0.23 vs. 0.67 ± 0.25 mmol·L^−1^; FDR < 0.05) as well as phospholipids (0.32 ± 0.14 vs. 0.43 ± 0.16 mmol·L^−1^; FDR < 0.001), cholesteryl esters (0.36 ± 0.15 vs. 0.41 ± 0.16 mmol·L^−1^; FDR < 0.05), triglycerides (0.42 ± 0.18 vs. 0.75 ± 0.38 mmol·L^−1^; FDR < 0.001), free cholesterol (0.20 ± 0.10 vs. 0.26 ± 0.10 mmol·L^−1^; FDR < 0.05), and lipids (1.31 ± 0.52 vs. 1.85 ± 0.72 mmol·L^−1^; FDR < 0.001) in VLDL were significantly lower in LEA compared to HEA. These differences were observed throughout all VLDL subclasses. Notable interactions between condition and state on these markers were found for average VLDL diameter and in triglycerides in HDL lipoproteins. Specifically, the difference in triglyceride concentrations in HDL between LEA and HEA was greater in EX compared to REST (FDR < 0.05), with lower concentrations found in LEA‐EX compared to LEA‐REST (42 ± 21 vs. 72 ± 14 μmol·L^−1^; Figure 4). Comprehensive subclass specific lipoprotein concentrations are provided in Table S1.

### 3.3 Prediction of LEA based on metabolic signature

**FIGURE 5:**
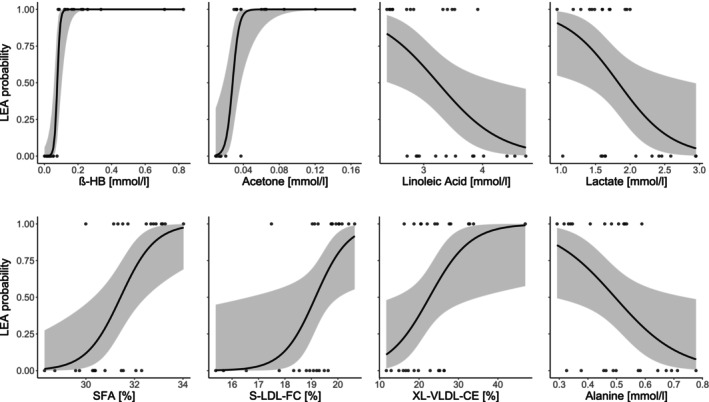
Probability of low energy availability (LEA) versus post‐intervention serum concentrations of β‐hydroxybutyrate (β‐HB), acetone, linoleic acid, lactate, the ratio of saturated fatty acids to total fatty acids (SFA%), free cholesterol to total lipids ratio in small LDL (S‐LDL‐FC %), cholesteryl esters to total lipids ratio in chylomicrons and extremely large VLDL (XL‐VLDL‐CE%), and alanine—Logistic regression analysis revealed β‐HB as the strongest predictor of LEA; LDL, low‐density lipoprotein; VLDL, very‐low‐density lipoprotein.

Within a logistic regression framework, eight out of the 234 metabolites or metabolite ratios were significant predictors of LEA (*p* < 0.05; Figure 5). These included acetone and β‐HB, the ratio of saturated fatty acids to total fatty acids (SFA%), linoleic acid, lactate, free cholesterol to total lipids ratio in small LDL (S‐LDL‐FC %), cholesteryl esters to total lipids ratio in chylomicrons and extremely large VLDL (XL‐VLDL‐CE%), and alanine. The model with the lowest AIC included only β‐HB, yielding robust model efficacy (pseudo *R* ^2^ = 0.880, *p* < 0.001, AIC = 8.318). LEA probability was ~1.0 above β‐HB concentrations of 0.09 mmol·L^−1^.

## 4 DISCUSSION

This is the first study to systematically identify changes in the human serum metabolome in response to controlled exposure to LEA. We employed high‐throughput NMR metabolomics to measure 250 metabolites and their ratios in serum blood samples obtained in two randomized controlled trials, in which trained individuals were exposed to LEA or HEA. Overall, we observed metabolomic changes indicative of increased use of fatty acids for energy metabolism and altered atherogenic profiles. B‐HB emerged as the best predictive biomarker of LEA, independent of whether LEA was achieved with or without exercise.

Exposure to LEA led to marked reductions in circulating fatty acids and triglycerides, a well‐documented response to energy restriction. These changes align with findings from other studies that demonstrate how short‐term LEA forces the body to mobilize fatty acids (Kojima et al., 2022), thereby decreasing triglycerides in circulation (Grundler et al., 2021; Jouhki et al., 2024; Lin et al., 2023; Mathew et al., 2014). Metabolic adaptations indicative of enhanced fat utilization and reduced reliance on carbohydrates were also highlighted by reductions in pyruvate, a key intermediate in energy metabolism, that may indicate reduced glycolytic activity. The reduced supply of Acetyl‐CoA by pyruvate may be counterbalanced by the increased breakdown of fatty acids (Castro et al., 2021; Leblanc et al., 2004).

Ketone body concentrations were significantly greater in LEA than in HEA. B‐HB particularly stood out and was increased 11.3‐fold in LEA when compared to HEA. This is not surprising, given the hepatic production of β‐HB during energy and carbohydrate deficiency, which serves to provide fuel for essential organs such as the heart and the brain (Madhavan & Stubbs, 2025) in the form of an alternative energy source to glucose (Huang et al., 2024). Consequently, it is well documented that β‐HB rises in response to low carbohydrate availability (Prins et al., 2023; Westman et al., 2002) and energy deficiency (Cox et al., 2016; Loucks, 2006; Loucks & Thuma, 2003; Loucks & Verdun, 1998; Solianik et al., 2023). The present study therefore confirms that short‐term LEA (3–5 days) with and without exercise substantially increases ketone body production. Moreover, the decreased serum triglycerides and fatty acids may also be attributed to the increased β‐HB concentrations, as energy and carbohydrate restriction stimulate lipolysis, leading to increased production of β‐HB (Han et al., 2020). Our data highlight β‐HB as a potential early biomarker for LEA; however, to our knowledge, its use as a specific biomarker for the detection of LEA in athletes or other populations is not yet established. As highlighted by Jouhki et al. (2024), ketone body recovery occurs differentially following a return to a high‐carbohydrate, iso‐energetic diet. Therefore, future studies are needed to confirm whether these characteristic changes persist over time. Although our logistic regression analysis showed that serum β‐HB concentrations above 0.09 mmol·L^−1^ perfectly predicted LEA in our controlled cohorts, this value should be interpreted cautiously due to the observed interindividual variability as well as potential discrepancies in metabolite concentrations between sampling sites (venous vs. capillary) and measurement technologies (NMR vs. point of care) (Norgren et al., 2020). Additionally, iso‐energetic carbohydrate‐restricting diets and exogenous ketone supplementation are potential confounders in free‐living settings, as both can elevate serum ketone concentrations to levels similar to or even higher than those observed within our study (Evans et al., 2017; Prins et al., 2023; Westman et al., 2002).

Contrary to β‐HB, acetoacetate displayed differential abundance across LEA conditions. Acetoacetate was significantly increased in LEA‐REST, but it was not detectable in LEA‐EX. The slightly higher β‐HB concentrations in LEA‐EX when compared to LEA‐REST might be due to a conversion of circulating acetoacetate into β‐HB during exercise via β‐HB‐dehydrogenase (Yurista et al., 2021). Elevated but limited metabolic clearance rate of ketone bodies into skeletal muscle (Evans et al., 2017) and higher ventilation rates during exercise (Dearlove et al., 2019) may also contribute to acetoacetate transformation and acetone clearance.

In addition to increased ketogenesis, energy deficits are believed to increase reliance on amino acid oxidation to meet energy demands (Carbone et al., 2014; Gwin et al., 2021). However, our data suggest that individual amino acids are differentially impacted by LEA. The most notable change in amino acid levels following short‐term LEA was a reduction in alanine. Since alanine is a glucogenic amino acid, this decrease could be attributed to its increased uptake by the liver, the primary site of gluconeogenesis (Sahoo et al., 2023). While the circulating levels of some amino acids decreased, others, such as BCAA, increased (Blackburn et al., 2020). It has been hypothesized that elevated BCAA arise from impaired BCAA oxidation in skeletal muscle (Kainulainen et al., 2013). Under conditions of energy deficiency, more BCAA are released due to increased proteolysis, but further breakdown in the muscle may be impaired due to the inhibition of the enzymes involved in BCAA degradation by way of reduced glycolysis and increased fatty acid oxidation (Holeček, 2020). It is important to acknowledge the impact of exercise on protein metabolism within our study population. It is well known that endurance exercise, such as that conducted in EX, can further increase protein turnover and oxidation (Knapik et al., 1991; Wagenmakers, 1998). For instance, there was no effect of condition on glutamine per se, but we observed significantly higher glutamine concentrations in REST when compared to EX.

Exercise further led to contrasting glycerol concentrations between EX and REST within LEA and HEA conditions. In EX, we observed 2.5 to 4.5‐fold higher serum glycerol concentrations when compared to REST, indicating a differential response when inducing LEA through dietary restriction alone or through a combination of reduced dietary energy intake and increased exercise energy expenditure, potentially leading to a redirection of energy fluxes during energy deficiency and exercise. The increased glycerol concentrations in EX, however, were not accompanied by increased concentrations of fatty acids, yet they suggest a possible increase in gluconeogenesis in a state of energy deficiency (Bortz et al., 1972; Robergs & Griffin, 1998).

Metabolic changes as evidence of altered energy metabolism were accompanied by improvements in the atherogenic risk profile, which included reductions in VLDL and related metabolites as well as in HDL‐triglyceride content (Girona et al., 2019). Similar findings have also been reported in response to caloric restriction in adults with overweight and obesity (Grundler et al., 2021) and in physique athletes undergoing intense weight loss during their competition preparation phase (Jouhki et al., 2024). In line with our observations, Jouhki et al. (2024) observed significant alterations in the VLDL lipidome, including reduced VLDL diameter, triglyceride levels, and lipid concentrations, accompanied by a decrease in GlycA in physique athletes losing on average 10.6 kg. Although their average rate of weight loss (~ 0.07 kg·d^−1^) was approximately nine times slower than in our short‐term experiments (~ 0.60 kg·d^−1^) and included continuous resistance and endurance training, Jouhki et al. (2024) reported similar metabolic adaptations over the course of several months, suggesting that metabolic changes in response to LEA are potentially stable over a longer timeframe. Furthermore, our results indicate that exercise amplifies the lipoprotein changes associated with LEA. For example, the HDL‐triglyceride concentrations lowering effect of LEA was much greater in EX compared to REST, possibly due to the increased oxidation of free fatty acids during exercise, which in turn can reduce the triglyceride content available for incorporation into HDL particles (Kraus et al., 2002; Romijn et al., 1993).

We also observed that the marked decrease in GlycA in response to LEA was modulated by the presence or absence of exercise. GlycA, a composite NMR‐based biomarker, has been closely linked to the leptin/adiponectin ratio and is indicative of adipose tissue dysfunction (Dullaart et al., 2015). Elevated GlycA levels have been associated with a pro‐inflammatory state and an increased risk of cardiovascular disease. In line with these associations, recent research suggests that weight loss, achieved either through diet alone or in combination with exercise, leads to a reduction in GlycA concentrations, indicating less inflammation and improved cardiometabolic health (Collins et al., 2023; Jouhki et al., 2024).

Although we are, to our knowledge, the first to report comprehensive metabolomics changes in response to controlled exposure to LEA, our study's findings must be contextualized within several methodological aspects that could influence the observed outcomes. Firstly, although our intervention protocols strictly controlled dietary intake, individual differences in the participants' habitual diet may have contributed to differences in changes in lipid profiles, as the influence of dietary fatty acids and carbohydrates on HDL and serum lipids is well‐documented (Mensink et al., 2003). All participants switched from their habitual diet to an all‐liquid study diet with fixed macronutrient ratios. In retrospect, we therefore consider the lack of a controlled diet leading into each intervention a limitation. However, we also feel that the liquid‐only diet is one of the big strengths of the study, by reducing noise that could have otherwise been introduced by differing dietary ingredients. This is also the reason why we opted to exclusively investigate post‐intervention data for the second part of our analysis of a “metabolic signature” of short‐term LEA. Secondly, our analysis combines data from two independently conducted studies (EX and REST), which were performed in the same laboratory but at different time points. Although both studies adhered to identical measurement protocols and utilized the same dietary products during their interventions, the lack of shared individual participants introduces potential variability. Thirdly, the duration of each condition varied between three (REST) and five (EX) days, which may have impacted the magnitude of change in metabolites. Although we acknowledge this discrepancy as a limitation, past research has shown that key endocrine regulators of energy balance such as leptin behave surprisingly similar even when the duration of LEA exposure varies (Koehler et al., 2016; Loucks & Thuma, 2003; Papageorgiou et al., 2018). Leptin acts as a mediator of the body's response to an energy deficit (Rosenbaum & Leibel, 2014) and is causally linked to metabolic adaptations during energy deficiency (Rosenbaum, 2005), highlighting its important role within human energy metabolism pathways. Therefore, we are confident that metabolic adaptations in both studies are generally comparable, regardless of the different exposure time to LEA. Finally, our analyses demonstrate that a series of metabolites (e.g., triglycerides, total fatty acids, β‐HB, and VLDL‐related metabolites) discriminate LEA from HEA. However, our findings remain associative and may be limited to adaptive metabolic alterations in response to acute energy deficiency. Even though many of our findings align with results from a longer‐term investigation (Jouhki et al., 2024), prospective and longitudinal studies are needed to determine the robustness of the reported metabolic signature during extended periods of LEA and for the prediction of downstream health outcomes.

## 5 CONCLUSION

Overall, our analysis contributes to the growing body of literature on LEA by highlighting its short‐term effects on the human metabolome. The identification of biomarkers through metabolomics offers a promising avenue for improving early identification, prevention, and management of LEA in athletic populations. Future research should focus on investigating the dose–response relationship between LEA and the responsive metabolites to better understand their sensitivity to varying levels of energy availability. Long‐term studies are needed to assess how these metabolomic changes persist over time and to explore how these markers recover after a return to normal energy availability. Further work should aim to refine the use of these biomarkers in early detection and prevention strategies.

## AUTHOR CONTRIBUTIONS

V.N. was involved in conceptualization, methodology, formal analysis, visualization, and writing—original draft. C.M. was involved in resources, investigation, formal analysis, and writing—review and editing. P.W. was involved in conceptualization and writing—review and editing. S.H., J.K.I., and J.J.H. were involved in writing—review and editing. A.B. was involved in writing—review and editing. K.K. was involved in conceptualization, writing—review and editing, and supervision. All authors approved the final manuscript. They agree to be accountable for all aspects of the work in ensuring that questions related to the accuracy or integrity of any part of the work are appropriately investigated and resolved. All persons designated as authors qualify for authorship, and all those who qualify for authorship are listed.

## FUNDING INFORMATION

Initial Data Collection and Sample Shipment were supported by institutional funding from the Technical University of Munich and the University of Nebraska‐Lincoln (awarded to K.K.). The analysis of the samples is supported by the Ministry of Education and Culture of Finland Grant (OKM/10/626/2021 and OKM/78/626/2022 to J.K.I.).

## CONFLICT OF INTEREST STATEMENT

All authors declare that they have no conflicts of interest.

## Supporting information


Figure S1.

Figure S2.

Table S1.

## Data Availability

Metabolomics data is accessible via DOI: 10.14459/2025mp1796657.
