# Supplementary material for: Metabolic signature of short‐term low energy availability
Source: Physiol Rep. 2025 Sep 29;13(19):e70582. doi: 10.14814/phy2.70582 (PMC12477441; doi:10.14814/phy2.70582)
Supplement: Supplementary file 1 — Figure S1. [file PHY2-13-e70582-s001.zip › Supplementary figure 1.docx]

**Supplementary figure 1**: Ranked mean metabolite change-ratios for low and high energy availability with exercise (LEA-EX vs. HEA-EX) for all metabolites, which showed a significant time × condition interaction effect (FDR < 0.05). Lipoprotein names include particle sizes (XXL, XL, L, M, S, XS), lipoprotein class declarations (VLDL = very low-density lipoprotein, LDL = low-density lipoprotein, IDL = intermediate density lipoprotein, HDL = high-density lipoprotein) and the indication of particle component (P = concentration, L = total lipids, PL = phospholipids, C = cholesterol, CE = cholesteryl esters, FC = free cholesterol, TG = triglycerides). “%” indicates a ratio of the respective component to Total Lipids. β-HB = β-hydroxybutyrate, BCAA = branched-chain amino acids, FA = Fatty acids, PUFA = poly-unsaturated FA, MUFA = mono-unsaturated FA, SFA = saturated FA, TG/PG = ratio of TG and phosphoglycerides, LA = linoleic acids, SEM = standard error of the mean.
