# Supplementary figures and images for: Metabolic signature of short‐term low energy availability
Source: Physiol Rep. 2025 Sep 29;13(19):e70582. doi: 10.14814/phy2.70582 (PMC12477441; doi:10.14814/phy2.70582)

# Metabolite Ratios (Post vs. Pre) in REST

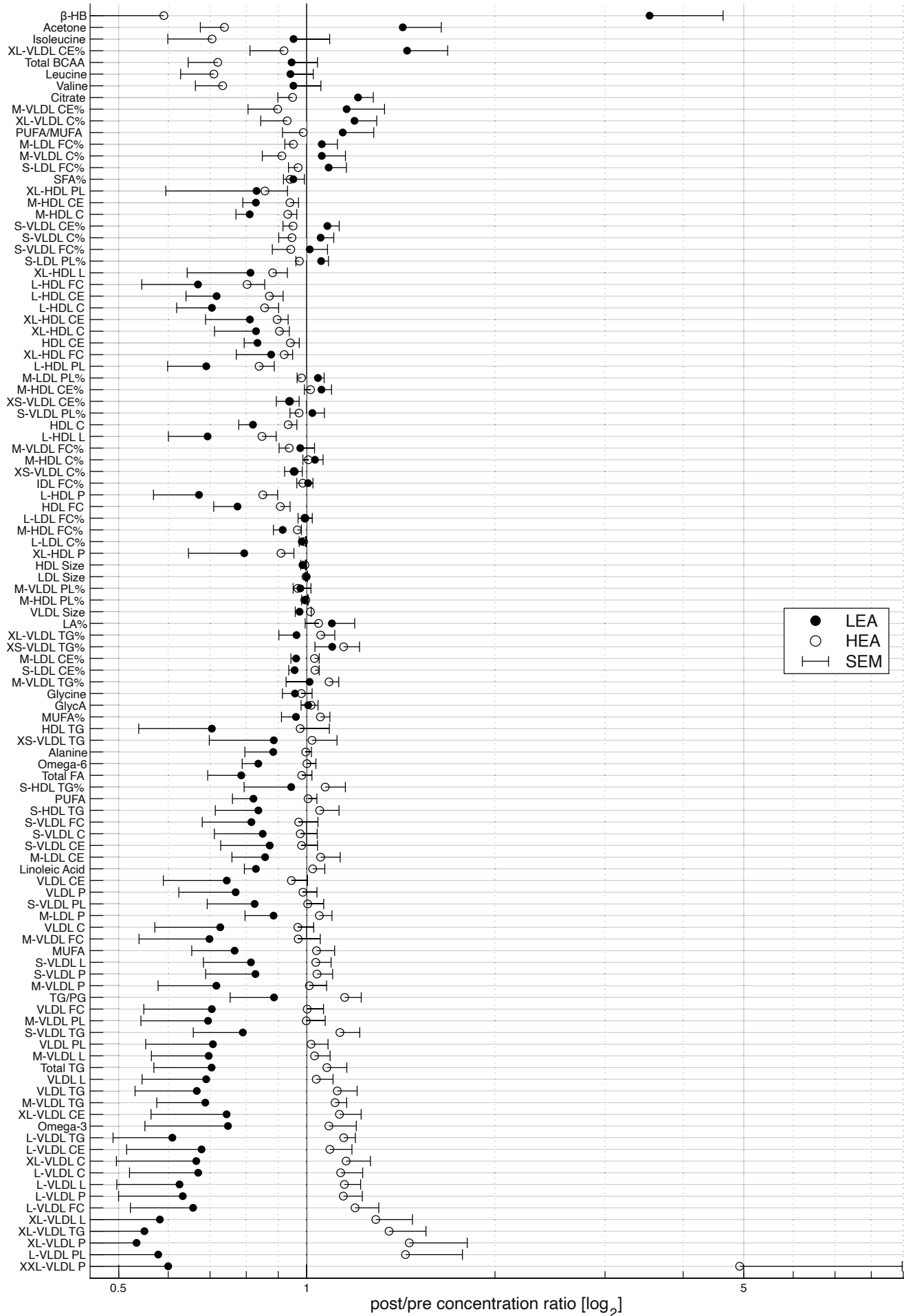

Supplement: Supplementary file 1 — Figure S1. [file PHY2-13-e70582-s001.zip › PHYSREP-2025-08-716-T-s02.pdf]

# Metabolite Ratios (Post vs. Pre) in EX

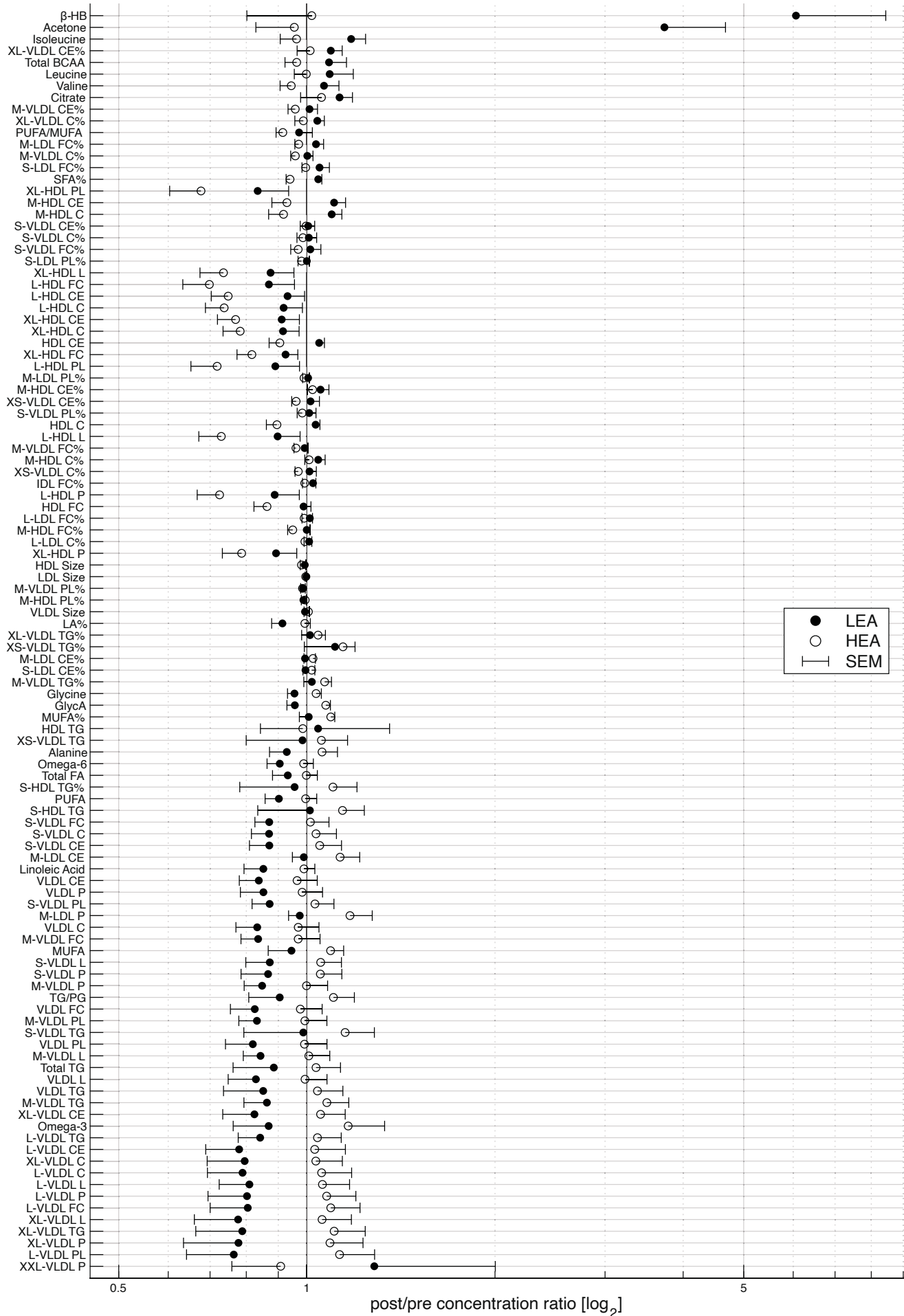

Supplement: Supplementary file 2 — Figure S2. [file PHY2-13-e70582-s002.zip › PHYSREP-2025-08-716-T-s03.pdf]
