# Supplementary material for: Metabolic signature of short‐term low energy availability
Source: Physiol Rep. 2025 Sep 29;13(19):e70582. doi: 10.14814/phy2.70582 (PMC12477441; doi:10.14814/phy2.70582)
Supplement: Supplementary file 3 — Table S1. [file PHY2-13-e70582-s003.docx]

***Supplementary table S1****: Post-intervention serum lipoprotein concentrations [µmol/l] of different lipoprotein sub-classes for LEA and HEA, with (EX) and without exercise (REST).*

|  | | **LEA** | | **HEA** | | *p-values (FDR)* | | |
| --- | --- | --- | --- | --- | --- | --- | --- | --- |
|  | | **EX (n=7)** | **REST (n=6)** | **EX (n=7)** | **REST (n=6)** | *P_C_* | *P_S_* | *P_CS_* |
| **XL-HDL** | TG | 3.4 ± 1.0 | 4.3 ± 1.3 | 6.3 ± 3.3 | 5.4 ± 1.6 | **< 0.001** | 0.441 | 0.441 |
|  | FC | 27.5 ± 3.6 | 26.5 ± 7.2 | 26.2 ± 3.8 | 21.3 ± 9.8 | 0.307 | **0.047** | 0.355 |
|  | CE | 65.8 ± 16.6 | 72.4 ± 29.9 | 63.3 ± 9.2 | 55.7 ± 39.4 | 0.729 | 0.402 | 0.703 |
|  | PL | 74.5 ± 27.8 | 93.9 ± 48.9 | 73.3 ± 14.3 | 70.1 ± 68.4 | 0.993 | 0.385 | 0.385 |
| **L-HDL** | TG | 4.7 ± 4.4 | 14.4 ± 7.4 | 16.2 ± 13.6 | 19.5 ± 9.2 | < **0.001** | 0.492 | **0.004** |
|  | FC | 56.3 ± 26.4 | 76.8 ± 41.5 | 57.3 ± 12.4 | 59.7 ± 52.5 | 0.648 | 0.648 | 0.648 |
|  | CE | 228.8 ± 94.6 | 284.0 ± 137.1 | 222.5 ± 39.9 | 228.0 ± 174.2 | 0.825 | 0.791 | 0.791 |
|  | PL | 228.5 ± 119.2 | 343.8 ± 165.2 | 236.8 ± 54.7 | 290.9 ± 204.9 | 0.637 | 0.838 | 0.637 |
| **M-HDL** | TG | 9.8 ± 8.3 | 23.4 ± 5.7 | 27.4 ± 19.8 | 38.0 ± 10.1 | < **0.001** | 0.079 | < **0.001** |
|  | FC | 56.5 ± 20.1 | 78.8 ± 22.9 | 60.4 ± 16.0 | 77.0 ± 22.0 | 0.520 | 0.238 | 0.998 |
|  | CE | 312.2 ± 85.7 | 397.8 ± 84.8 | 311.7 ± 57.8 | 389.5 ± 76.2 | 0.770 | 0.069 | 0.770 |
|  | PL | 311.0 ± 84.3 | 419.5 ± 75.1 | 336.3 ± 70.7 | 438.0 ± 74.7 | 0.319 | **0.011** | 0.960 |
| **S-HDL** | TG | 24.3 ± 8.5 | 30.3 ± 8.4 | 38.9 ± 16.9 | 43.2 ± 13.5 | < **0.001** | 0.510 | **0.026** |
|  | FC | 92.9 ± 10.9 | 102.2 ± 10.9 | 99.5 ± 15.9 | 104.9 ± 8.0 | 0.454 | 0.454 | 0.974 |
|  | CE | 305.7 ± 34.8 | 312.5 ± 24.3 | 302.3 ± 30.6 | 323.0 ± 35.0 | 0.923 | 0.189 | 0.189 |
|  | PL | 475.7 ± 59.1 | 558.0 ± 42.5 | 502.7 ± 69.0 | 604.0 ± 56.2 | 0.241 | **0.001** | 0.391 |
| **IDL** | TG | 70.2 ± 14.3 | 76.6 ± 12.8 | 98.1 ± 36.5 | 78.0 ± 9.3 | < **0.001** | 0.184 | 0.053 |
|  | FC | 236.3 ± 57.4 | 192.8 ± 50.2 | 243.8 ± 42.6 | 154.5 ± 30.5 | 0.579 | < **0.001** | 0.579 |
|  | CE | 612.5 ± 145.4 | 533.8 ± 146.4 | 651.7 ± 122.9 | 458.5 ± 89.6 | 0.340 | **0.004** | 0.805 |
|  | PL | 297.3 ± 68.5 | 252.3 ± 67.4 | 321.2 ± 59.4 | 213.8 ± 38.0 | 0.181 | < **0.001** | 0.456 |
| **L-LDL** | TG | 75.5 ± 14.8 | 78.1 ± 11.7 | 102.1 ± 38.0 | 78.1 ± 10.0 | < **0.001** | 0.086 | **0.047** |
|  | FC | 340.8 ± 83.6 | 276.0 ± 75.3 | 344.3 ± 51.8 | 231.3 ± 40.0 | 0.970 | < **0.001** | 0.970 |
|  | CE | 900.8 ± 226.8 | 744.5 ± 224.5 | 918.2 ± 146.3 | 661.3 ± 142.3 | 0.790 | **0.006** | 0.790 |
|  | PL | 387.2 ± 90.2 | 318.8 ± 75.8 | 403.8 ± 63.1 | 285.3 ± 53.5 | 0.504 | < **0.001** | 0.817 |
| **M-LDL** | TG | 24.2 ± 5.1 | 25.0 ± 4.1 | 33.9 ± 14.0 | 28.0 ± 6.2 | < **0.001** | 0.240 | 0.208 |
|  | FC | 141.0 ± 34.3 | 112.9 ± 29.3 | 141.2 ± 19.8 | 98.0 ± 21.6 | 0.976 | **0.001** | 0.976 |
|  | CE | 308.0 ± 82.4 | 248.0 ± 77.2 | 346.5 ± 74.6 | 276.0 ± 112.3 | 0.106 | 0.115 | 0.866 |
|  | PL | 175.5 ± 42.6 | 143.8 ± 37.2 | 186.8 ± 32.4 | 138.8 ± 37.4 | 0.291 | **0.040** | 0.999 |
| **S-LDL** | TG | 9.9 ± 2.2 | 10.3 ± 2.5 | 14.8 ± 6.1 | 13.8 ± 4.7 | < **0.001** | 0.609 | 0.474 |
|  | FC | 62.0 ± 12.1 | 50.5 ± 10.9 | 60.2 ± 7.1 | 43.1 ± 7.0 | 0.450 | < **0.001** | 0.977 |
|  | CE | 139.3 ± 32.0 | 113.3 ± 29.2 | 154.8 ± 29.1 | 117.4 ± 36.9 | 0.064 | **0.047** | 0.999 |
|  | PL | 101.1 ± 16.4 | 85.7 ± 16.8 | 103.3 ± 14.0 | 77.2 ± 11.2 | 0.748 | < **0.001** | 0.748 |
| **XXL-VLDL** | TG | 34.9 ± 28.7 | 27.1 ± 45.3 | 91.4 ± 61.2 | 122.4 ± 126.0 | **0.007** | 0.864 | 0.336 |
|  | FC | 6.7 ± 6.4 | 5.5 ± 8.8 | 18.5 ± 12.3 | 21.9 ± 21.6 | **< 0.001** | 0.769 | 0.769 |
|  | CE | 14.9 ± 9.8 | 7.0 ± 10.4 | 32.4 ± 19.7 | 25.9 ± 24.9 | **0.002** | 0.282 | 0.841 |
|  | PL | 7.6 ± 9.4 | 6.4 ± 12.4 | 26.2 ± 19.9 | 32.6 ± 33.5 | **0.001** | 0.721 | 0.721 |
| **XL-VLDL** | TG | 36.5 ± 19.0 | 35.4 ± 40.3 | 72.2 ± 38.5 | 108.3 ± 87.5 | < **0.001** | 0.721 | 0.474 |
|  | FC | 12.9 ± 7.4 | 8.8 ± 9.8 | 21.0 ± 9.1 | 20.7 ± 17.7 | < **0.001** | 0.473 | 0.473 |
|  | CE | 26.0 ± 11.7 | 15.9 ± 12.4 | 35.7 ± 12.1 | 24.4 ± 17.6 | < **0.001** | 0.138 | 0.961 |
|  | PL | 18.8 ± 12.2 | 12.8 ± 16.1 | 32.8 ± 15.6 | 34.4 ± 31.1 | < **0.001** | 0.482 | 0.183 |
| **L-VLDL** | TG | 80.0 ± 22.7 | 80.7 ± 55.7 | 115.1 ± 34.3 | 178.8 ± 119.2 | < **0.001** | 0.479 | 0.299 |
|  | FC | 26.7 ± 13.5 | 21.2 ± 18.4 | 40.6 ± 15.6 | 42.3 ± 31.8 | < **0.001** | 0.642 | 0.642 |
|  | CE | 36.8 ± 18.0 | 25.7 ± 20.3 | 50.3 ± 16.4 | 38.8 ± 26.8 | < **0.001** | 0.330 | 0.668 |
|  | PL | 32.5 ± 19.0 | 27.0 ± 27.9 | 55.1 ± 24.1 | 61.9 ± 48.2 | < **0.001** | 0.792 | 0.558 |
| **M-VLDL** | TG | 149.7 ± 40.0 | 155.9 ± 79.0 | 211.8 ± 65.6 | 273.0 ± 145.0 | < **0.001** | 0.548 | 0.548 |
|  | FC | 75.4 ± 29.2 | 53.4 ± 28.3 | 88.4 ± 22.5 | 55.0 ± 26.3 | 0.061 | 0.059 | 0.819 |
|  | CE | 114.4 ± 42.2 | 76.8 ± 38.0 | 125.2 ± 29.1 | 58.2 ± 20.4 | 0.280 | < **0.001** | 0.757 |
|  | PL | 114.6 ± 43.9 | 84.6 ± 45.2 | 139.8 ± 37.3 | 97.9 ± 48.9 | **0.030** | 0.118 | 0.707 |
| **S-VLDL** | TG | 68.1 ± 19.9 | 89.6 ± 33.2 | 113.8 ± 50.8 | 140.6 ± 49.6 | < **0.001** | 0.331 | 0.625 |
|  | FC | 58.3 ± 19.3 | 43.8 ± 18.2 | 67.6 ± 15.4 | 41.1 ± 13.3 | **0.034** | **0.008** | 0.703 |
|  | CE | 91.5 ± 33.6 | 67.1 ± 29.3 | 111.1 ± 28.6 | 64.4 ± 21.1 | **0.006** | **0.006** | 0.366 |
|  | PL | 87.2 ± 27.7 | 69.2 ± 27.7 | 106.0 ± 26.4 | 72.1 ± 24.2 | **0.005** | **0.043** | 0.726 |
| **XS-VLDL** | TG | 38.7 ± 10.6 | 47.2 ± 10.8 | 60.2 ± 26.4 | 53.7 ± 7.9 | < **0.001** | 0.681 | 0.070 |
|  | FC | 50.8 ± 14.1 | 43.2 ± 12.9 | 59.5 ± 14.8 | 36.9 ± 7.7 | **0.007** | **0.003** | 0.234 |
|  | CE | 118.7 ± 33.1 | 97.0 ± 30.4 | 132.5 ± 30.5 | 78.1 ± 18.8 | 0.167 | < **0.001** | 0.417 |
|  | PL | 88.7 ± 24.1 | 78.9 ± 23.2 | 109.4 ± 29.5 * | 69.5 ± 13.1 | < **0.001** | **0.005** | 0.176 |
| *Values are displayed as mean ± standard deviation. FDR-corrected p-values indicate effects of condition (P_C_), state (P_S_) and their interaction (P_CS_). Lipoprotein names include particle sizes (XXL, XL, L, M, S, XS), lipoprotein class declarations (VLDL = very low-density lipoprotein, LDL = low-density lipoprotein, IDL = intermediate density lipoprotein, HDL = high-density lipoprotein) and the indication of particle component (TG = triglycerides, FC = free cholesterol, CE = cholesteryl esters, PL = phospholipids)* | | | | | | | | |
